# *Trichoderma koningiopsis* Tk905: an efficient biocontrol, induced resistance agent against banana Fusarium wilt disease and a potential plant-growth-promoting fungus

**DOI:** 10.3389/fmicb.2023.1301062

**Published:** 2023-11-07

**Authors:** Mei Luo, Yue Chen, Qiurong Huang, Zhenxin Huang, Handa Song, Zhangyong Dong

**Affiliations:** ^1^Innovative Institute for Plant Health, Zhongkai University of Agriculture and Engineering, Guangzhou, China; ^2^Key laboratory of Fruit and Vegetable Green Prevention and Control in South-China, Ministry of Agriculture and Rural Affairs, Guangzhou, China

**Keywords:** *Trichoderma koningiopsis*, soil-borne disease, induced resistance, plant-growth promoting, biocontrol

## Abstract

*Fusarium oxysporum* f. sp. *cubense* tropical race 4 (FocTR4) is a devastating phytopathogen responsible for significant losses in banana production worldwide. *Trichoderma* and other biocontrol agents (BCAs) have been used as suitable disease control methods for banana Fusarium wilt. In this study, the endophytic *T. koningiopsis* Tk905 strain was isolated from the roots of dendrobe plants and identified utilizing morphological and molecular analyses. Antifungal activity tests revealed that Tk905 effectively inhibited mycelial growth with inhibition rates ranging from 26.52 to 75.34%. Additionally, Tk905 covered the pathogen mycelia, and spores were observed on or around the pathogen hyphae. The average root and shoot fresh weights and plant height, of Tk905-inoculated plants were significantly higher than those of the untreated plants. Furthermore, Tk905 treatment significantly increased the activity of antioxidant enzymes, such as catalase (CAT), phenylalanine ammonia-lyase (PAL), polyphenol oxidase (PPO), and peroxidase (POD), suggesting that Tk905 may enhance plant defence systems by activating their antioxidant mechanisms. Most importantly, Tk905-treated plants inoculated by three methods exhibited significantly lower disease incidence and severity than untreated plants. The protective effects of Tk905 against FocTR4 infection were not only observed in the early stages of infection but persisted throughout the experiment, suggesting that *T. koningiopsis* Tk905 can provide long-lasting protection against Fusarium wilt.

## Introduction

Banana (Musa spp.) is the second largest tropical and subtropical region fruit crops. According to the Food and Agriculture Organization of the United Nations (FAO, http://www.faostat.fao.org/) in 2017, bananas were planted in more than 130 countries worldwide, covering an annual planting area of more than 5 million hm^2^. China is the second largest banana producer and the largest banana consumer worldwide.

Banana diseases are important constraints in banana production. The soil-borne fungus *Fusarium oxysporum* f. sp. *cubense* (Foc) triggers Fusarium wilt, one of the most devastating diseases seriously threatening banana production worldwide (Ploetz, 2015). Foc tropical race 4 (FocTR4) can infect almost all banana cultivars (Kema and Weise, 2013; Ploetz, 2015) and has spread all over the world (Kema and Weise, 2013; Kema, 2019). Although much effort has been made to improve host resistance, the available Fusarium wilt resistant varieties are still limited and no suitable control management strategy for FocTR4 exists. Another approach to control soil-borne pathogens such as FocTR4 is the recently introduced Rhizosphere immunity method (Shen et al., 2015; Wei et al., 2020).

*Trichoderma* (Hypocreales: Ascomycota) is a filamentous fungal genus consisting of diverse microorganisms of agricultural importance due to their beneficial properties and multiple modes of antifungal action. Among environment-friendly biological control products used against pathogens, Trichoderma are the most efficient, convenient, and common (De Rezende et al., 2020; Tao et al., 2023). Trichoderma species are widely distributed in the soil, plant residues, endophytes, and other habitats and have been found to be effective in controlling many pathogens, including Fusarium spp. (Galletti et al., 2020; Karuppiah et al., 2022; Tao et al., 2023). Recently, considerable research has been focusing on Trichoderma plant growth promoting properties and their role in the systemic acquired resistance (SAR) and induced systemic resistance (ISR) of plants against phytopathogens (Karuppiah et al., 2022; Salwan et al., 2022).

Under this light, the main objectives of this study were to: (1) screen for endophytic *Trichoderma* strains, (2) identify the isolated Tk905 endophytic strain, (3) evaluate its plant growth promoting effects, and (4) investigate Tk905’s antifungal activity and induced resistance.

## Materials and methods

### Plant material, pathogen isolation, and inoculation

The highly aggressive *Fusarium oxysporum* f. sp. *cubense* tropical race 4 (FocTR4) 14013 strain used in this study was isolated from the diseased roots and stems of the Cavendish banana (Musa spp. AAA group) and identified (Liu et al., 2023). This pathogenic strain was kept at 4–10°C and −80°C in both the Culture Collection of Innovative Institute for Plant Health, Zhongkai University of Agriculture and Engineering, Guangdong Province, China and Guangdong Microbial Culture Collection Centre (GDMCC, 61934).

Cavendish banana plants (Musa spp. AAA group) were used in this study. Healthy banana tissue culture seedlings were transplanted into 90 mm diameter pots using a sterile peat-coconut coir mix. The seedlings were randomly distributed in a culture chamber at 28°C with a 16 h light/8 h dark photoperiod. After around 8 weeks, plants with five leaves and a healthy root system were chosen for Foc inoculation, which was undertaken as described by Dong et al. (2020), with minor modifications. Plant roots were soaked for 30 min in a 2 L spore suspension containing 10^7^ conidia/mL of the 14,013 FocTR4 strain and then planted in pots.

### Isolation of Trichoderma spp.

Five dendrobe plants were collected from a farm in Panyu, Guangzhou, China (22°93’N, 113°38′E). All samples were stored at 4°C in the refrigerator until endophyte isolation. Isolation was performed within 48 h of collection according to the method described by Dong et al. (2021). Healthy dendrobe roots were initially washed with running tap water for several minutes and then with sterile water for three times. The roots were cut into about 3 mm-long pieces and each piece was then surface sterilized by being dipped into 75% ethanol for 30 s and 2.5% sodium hypochlorite (NaClO) for 20–60 s, before being rinsed three times with sterilized water. Then the cuttings were dried on sterilized filter paper and placed on the surface of Trichoderma selective medium (TSM: MgSO_4_·7H_2_O 0.2 g; K_2_HPO_4_ 0.9 g; KC1 0.15 g; NH_4_NO_3_ 1.0 g; glucose 3.0 g; chloramphenicol 0.25 g; rose-bengal 0.15 g; agar 20 g with 1 L water) plates. The spread plate method was used to test whether the sterilized water of the last rinse contaminated by other fungi or not (Dong et al., 2021). Finally, the plates were incubated at 28°C for 2–3 d. Colonies growing on TSM were then transferred to the water agar (WA) plates for single spore isolation. After that, the isolates were cultured at potato dextrose agar (PDA) plates. Single spore isolates were stored at 4°C until further use in this study.

## Identification of *Trichoderma* Tk905

### Molecular identification

Total genomic DNA was extracted from mycelia grown on PDA and incubated for approximately 5–7 d at 28°C using the modified cetyltrimethylammonium bromide (CTAB) method. Primers ITS5 (5’-GGAAGTAAAAGTCGTAACAAGG-3′) and ITS4 (5’-TCCTCCGCTTATTGATATGC-3′) were used to amplify the rDNA ITS regions (White et al., 1990). Primers EF1-728F (5’-CATCGAGAAGTTCGAGAAGG-3′, Carbone et al., 1999), and EF1-986R (5’-TACTTGAAGGAACCCTTACC-3′, Jaklitsch et al., 2005) were used to amplify the translation elongation factor 1 (TEF1) gene. The amplicons were visualized using 1.5% agarose electrophoresis gel. Positive amplicons were sequenced by Tianyi Huiyuan Biotechnology Co., Ltd., Guangzhou, China.

### Phylogenetic analysis

The sequences obtained in the present study were checked by BioEdit v 7.25 (Hall, 2006) and blast in the NCBI^1^ by using the blastn. For the phylogenetic analysis, reference sequences for *Trichoderma* species and related taxa were obtained from the NCBI GenBank database. Each locus was aligned using the MAFFT^2^ (Katoh et al., 2019). The alignment results were trimmed using the Trimal tool and then combined by the PhyloSuite (v1.2.1). Phylogenetic analyses were runned based on maximum likelihood (ML) in RAxML v. 8.2.12 (Stamatakis, 2014), maximum parsimony (MP) in PAUP (v.4.0b10) (Swofford, 2002) and Bayesian analysis (BI) in MrBayes (v. 3.0b4) (Ronquist and Huelsenbeck, 2003).

### Screening of antagonistic isolates

The dual culture technique was used to screen isolates with antagonistic activity against *FocTR4* 14,013 strain and other pathogens, such as *Alternaria alternata*, *Calonectria ilicicola* (Chen et al., 2022), *Colletotrichum gloeosporioides*, *F. oxysporum* from *Morinda officinalis* (Dong et al., 2019), *Helminthosporium maydis*, *Lasiodiplodia pseudotheobromae* (Chen et al., 2020), and *Stagonosporopsis pogostemonis* (Luo et al., 2022). All of the strains were cultured by PDA plates at 28°C. A 5 mm in diameter disc from the margin of a 3 days-old *Trichoderma* colony was placed on one side of a PDA plate (8.5 cm diameter) together with another 5 mm disc from freshly cultured mycelia of pathogens placed on the opposite side with a 5 cm diameter distance. Each treatment was replicated five times.

After an incubation period of 7 days at 28°C, the growth inhibition of the pathogen colony was calculated in comparison to the control, according to the following formula: Ig (%) = $\frac{Cg-Tg}{Cg-0.5}$ × 100, where Ig (%) is the percentage inhibition of radial mycelial growth, *C*g is the radius length (mm) of the pathogen colony in the control, and *T*g is the radius length (mm) in the dual-culture. Mean percentage ± standard error of the mean (*n* = 5) was calculated.

### Inoculation of Cavendish banana seedlings with *Trichoderma* strain Tk905

The banana tissue culture seedlings were bought from company and then cultured with sterile matrix soil. Four leaf stage healthy banana seedlings with fresh weights of approximately 6 g were selected for the experiments. *Trichoderma* isolate Tk905 was cultured on PD for 7–10 days with the 180 rpm/min at 28°C. The mixture were then centrifuged at 10000 rpm/min at 4°C to collect the mycelium and spores. For each seedling, a total of 10 g mycelium and spores were collected from cultures, washed with sterile water three times and transferred in a container with 200 mL of sterile water, then mixed with soil. Finally, an additional 250 mL of H_2_O was added in both with and without *Trichoderma* soil. This mixture was maintained for 5 days, and subsequently was added H_2_O daily to the soil. Then after 5 days, the banana seedlings were transplanted to the soils with the fungal suspension (T+ treatment, with *Trichoderma* strain Tk905) or with water in control plants (T−, without *Trichoderma* strain Tk905). Each pot was watered and cultured under natural conditions at approximately 28–32°C temperature. Ten seedlings were used for each treatment. All experiments were performed in triplicate.

After 26 d of growth, root length, root weight, crown weight, plant height, ball diameter, crown dry weight, and root dry weight were measured. Each pot was submerged in water for 30 min at room temperature, and the plants were lightly shaken until the substrate was completely removed to avoid root loss or damage during harvesting. The roots were then rinsed with tap water until all visible sand and soil particles were removed.

### Enzymatic assay

The root system was inoculated in T+ and control plants (T−) as described in the previous section. 1 g of *Cavendish banana* leaves tissue and roots from various treatments (T+, T−) in different time sampling (0, 12, 24, 48, and 72 h after *Trichoderma* strain Tk905 inoculation) were homogenized using 5 mL ice-cold 100 mM phosphate buffer (pH 6.4) containing 0.5 polyvinyl pyrrolidone (PVP) and centrifuged at 6000 rpm for 50 min at 4°C. The activity of polyphenol oxidase (PPO) was determined by NBT reduction method (Anderson and Morris, 2011). The peroxidase (POD) was determined by guaiacol method (Ciou et al., 2011). For catalase (CAT) extraction, a 0.05 M phosphate buffer (pH 7.0) was used following Naeem-Abadi and Keshavarzi (2016). The phenylalanine ammonia-lyase (PAL) was detected according to Martinez-Tellez et al. (1998). All enzymatic activity data are presented as the mean of three individual samples at each time point. Total soluble protein content was determined according to Bradford (1976), using bovine serum albumin as a standard.

### Fungicide compatibility tests

The compatibility of *T. koningiopsis* Tk905 with commercially available fungicides against *FocTR4* 14,013 was determined. Seven fungicides containing mefentrifluconazole + pyraclostrobin, pyraclostrobin + fluxapyroxad, fluopyram + trifloxystrobin, propiconazole + azoxystrobin, difenoconazole + azoxystrobin, pydiflumetofen + difenoconazole, and difenoconazole were selected for compatibility tests. Each fungicide was tested at their recommended doses using a dilution method. Warm PDA around 60–65°C was mixed with each fungicide and poured into a sterile Petri dish to obtain the final concentrations of its recommended dose. Trichoderma plates were cultured on PDA for 7 d at 28 ± 2°C and a mycelial plug of 0.5 cm in diameter from each fungus was placed in the centre of prepared PDA plates and incubated for 3 days at 28 ± 2°C. The growth inhibition was calculated in comparison with the control.

### *In vivo* biological control of FocTR4 by *Trichoderma* strain Tk905

The experiment was arranged at Zhongkai university of agriculture and engineering, South China. The *Cavendish banana* was used to demonstrate the efficacy of *T. koningiopsis* Tk905 in controlling banana Fusarium disease caused by FocTR4. The experiments were conducted on May 2020 and 2022. Five leaf stage *Cavendish banana*, aged about 2 months, were transplanted and planted in 9 cm-diameter pots, with nutrient soil (vermiculite: soil = 1:4). The experiment was conducted using a completely randomized design (CRD) with 10 plant seedling replicates. The entire experiment was conducted in triplicate. The treatments included three different types of inoculation: (1) banana plants inoculated only with FocTR4 spores (T-F+); (2) banana plants co-inoculated mycelium and spores of Tk905 + T + F and FocTR4 spores by root irrigation, mix with soil and spray (T+F+); (3) *FocTR4* inoculated banana plants treated with pydiflumetofen + difenoconazole (PD + F+); (4) co-inoculated banana plants with Tk905 and *FocTR4* (Mix soil), treated with pydiflumetofen + difenoconazole (T + PD + F+); (5) non-inoculated plants (T-F-). Tk905 was added 24 h prior to *FocTR4* inoculation. A total of 8 mL Tk905 was put around the roots using by sterile pipette for the root irrigation experiment. After 10 days, a total of 25 mL FocTR4 spore suspension (3 × 10^7^/mL) was incubated around the roots. The mix with soil method was using the Tk905 cultured by solid-state fermentation. 3 g Tk905 fermentation was mixed with soil. Using the manual mini spray to spray the front and back of banana leaves was used for the spray method. The incidence rate and disease index were recorded 45 d after inoculation using the following formulas.

$$\begin{array}{l} \mathrm{Disease index}=\\ \Sigma \;\left(\mathrm{number of leaves in each disease grade}\times \mathrm{grade value}\right)/\\ \left(\mathrm{total number of assessed leaves}\times \mathrm{highest grade value}\right). \end{array}$$

$$\begin{array}{l} \mathrm{Disease inhibition rate}\;\left(\%\right)=\\ \left(\mathrm{disease index of control}-\mathrm{disease index after treatment}\right)/\\ \mathrm{disease index of control}\times 100. \end{array}$$

### Statistical analysis

All data were statistically evaluated using ANOVA and means were separated by the Duncan’s multiple range test (*p* < 0.05 probability) using the Data Processing System (DPS, Tang and Zhang, 2013). Data were presented as mean ± standard error (SE). Graphs were constructed using Microsoft Excel.

## Results

### Morphological and molecular identification of the *Trichoderma* Tk905 strain

**Figure 1:**
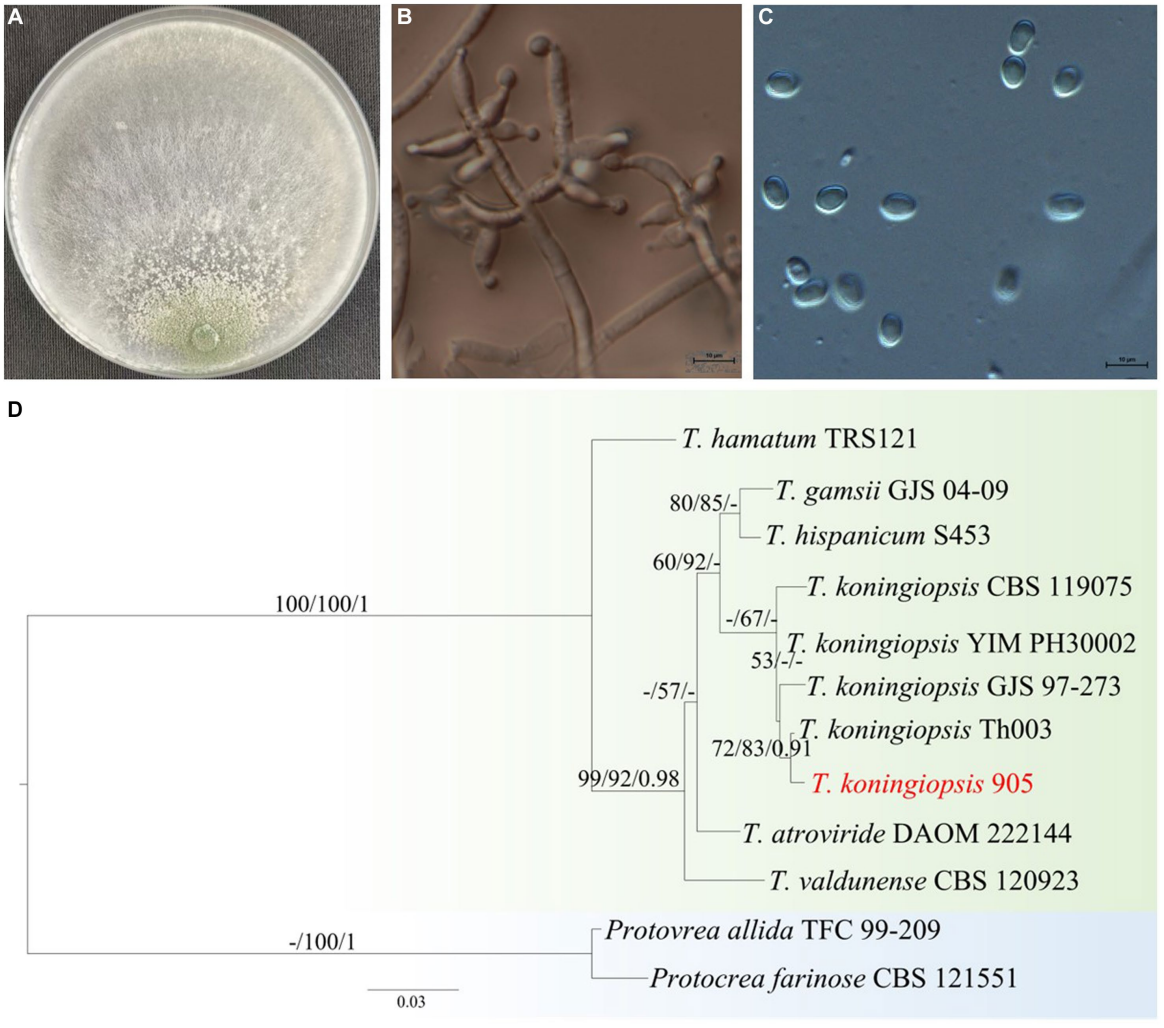
The morphological characteristics and phylogenetic tree of stain Tk905. **(A)** The colony on PDA medium. **(B)** Conidiophore and conidia. **(C)** Conidia. **(D)** phylogenetic tree. Scale bars in B and C are 10 μm.

The endophyte *Trichoderma* Tk905 strain isolated from the roots of the plant exhibited typical morphological features of *T. koningiopsis* based on the monograph of Samuels et al. (2006). Conidia were distributed on the aerial hyphae and eventually acquired a green later color. Conidial cells were oval to long oval, had smooth walls and a size of 2.7–5.3 μm × 2.0–3.5 μm (3.6 ± 0.6 × 2.7 ± 0.4 μm). Conidiophore upright, about 2 μm wide, with an obvious main axis. Conidiophores produced bottled stems or secondary branches directly. The angle between the branches and the main axis was slightly less than 90°. The tops of the fertile branches produced 2–5 bottle stems arranged in a vortex. Chlamydospores were not observed. The colony was initially white and after 2–3 days on PDA medium it started to produce spores and became lawn-like from the middle, with no obvious odor (Figures 1A–C).

The ITS sequence length of strain Tk905 was 604 bp (GenBank accession number OR083438). Tk905 sequence was up to 100% identical to known *T. koningiopsis* sequences belonging to strains CBS119075, MT111912, MN602617, MF616361, MH512944, KY111266, KC884758, EU718083, and DQ379015. The Tk905 Tef1-α sequence was 354 bp long (GenBank accession number: OR096250).

Phylogenetic analysis based on ITS and TEF-1α genes revealed that the Tk905 strain shared the highest homology in the phylogeny and clustered together with *T. koningiopsis* strains (Figure 1D). Based on the morphological and molecular data, the Tk905 strain was identified as *T. koningiopsis* and named *T. koningiopsis* Tk905.

### Antifungal of *Trichoderma* strain Tk905 against FocTR4

**Figure 2:**
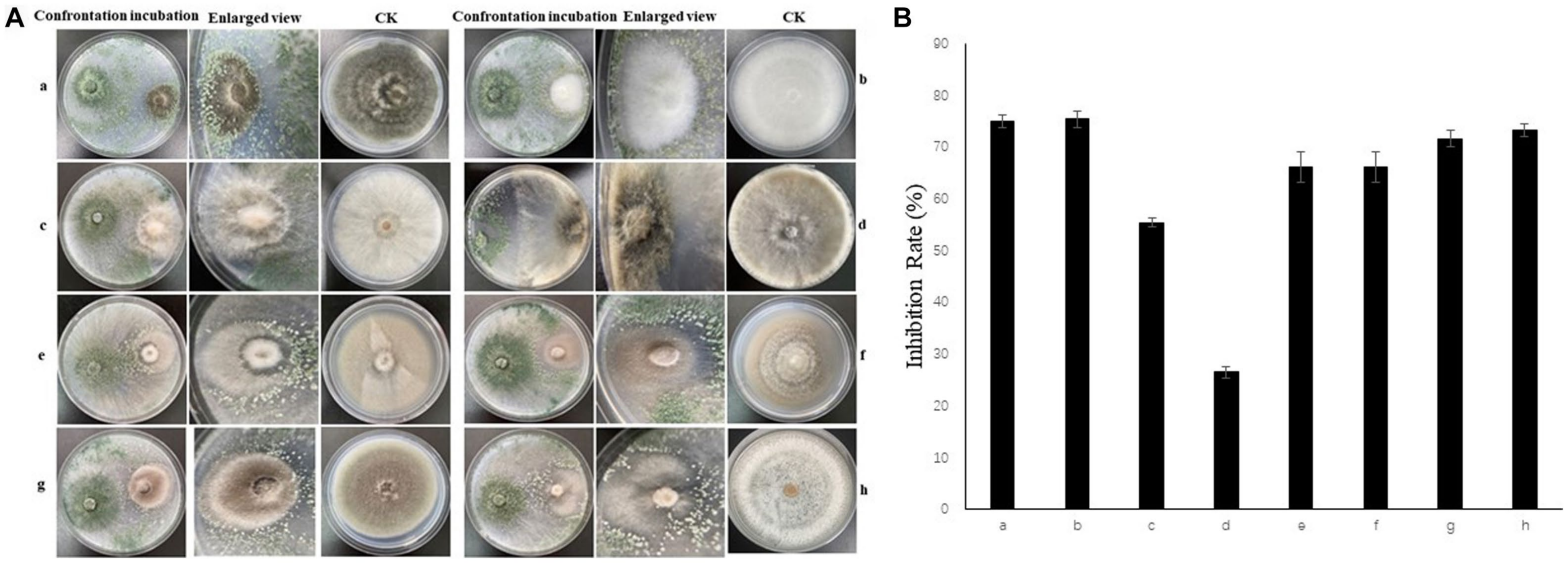
*In vitro* dual culture assay of Tk905 against different pathogenic fungi on PDA medium at 7 d. **(A)** Inoculated with pathogenic fungi. **(B)** Inhibitor rate. **(a)** *Helminthosporium maydis*. **(b)** *Fusarium oxysporum*. **(c)** *Calonectria ilicicola*. **(d)** *Lasiodiplodia pseudotheobromae*. **(e)** *Alternaria alternata*. **(f)** *Stagonosporopsis pogostemonis*. **(g)** *Fusarium oxysporum* f. sp. *cubense* tropical race 4. **(h)** *Colletotrichum gloeosporioides*. The CK stands for each pathogen tested in the dural cultures. Data represent the mean of five replicates. Error bars indicate the standard error (SE).

In this study, Tk905 was cultured with *A. alternata*, *C. ilicicola*, *Co. gloeosporioides*, *F. oxysporum*, *FocTR4* 14,013, *H. maydis*, *L. pseudotheobromae*, and *S. pogostemonis* for 7 days and was able to inhibit them in varying degrees. The Tk905 mycelial growth inhibition rates ranged between 26.52–75.34% (Figure 2B). The Tk905 mycelium covered the pathogen mycelium and some Tk905 spores were observed on or around the pathogen hyphae (Figure 2A).

### *Trichoderma* Tk905 banana plant growth promoting properties

**Figure 3:**
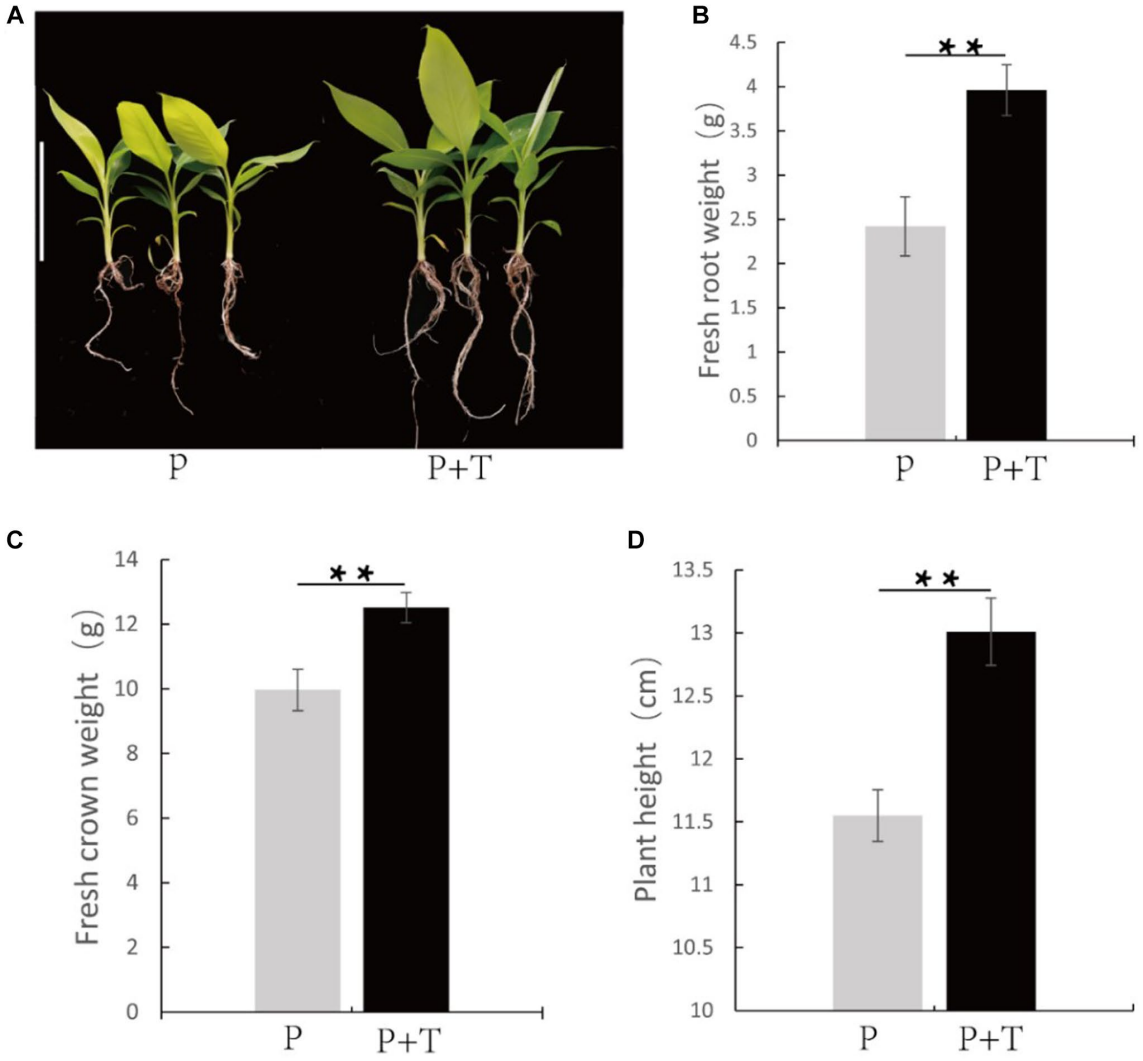
*Trichoderma koningiopsis* Tk905 improves the banana plants’ growth. **(A)** Banana treated with and without *T. koningiopsis* Tk905 after 26 d. **(B)** Root fresh weight (G). **(C)** Shoot fresh weight (g). **(D)** Root length (CM). Data represent the mean of five replicates. P + T means the banana plants treated with Tk905, and P means the banana plants only. Error bars indicate the standard error (SE, **p < 0.01, Student’s *t*-test).

Furthermore, growth-promoting effects of *T. koningiopsis* Tk905 were evaluated based on fresh root height, plant height, and fresh weight measurements of the banana plants. The average root fresh weight, shoot fresh weight and plant height of the *T. koningiopsis* Tk905-inoculated plants were 3.96 ± 0.29 g, 12.51 ± 0.47 g, and 13.01 ± 0.27 cm, which were significantly higher than those of untreated plants with 2.42 ± 0.33 g, 9.96 ± 0.64 g, and 11.55 ± 0.21 cm, respectively (*p* < 0.05). Overall, the Tk905 strain significantly affected root fresh weight (38.89%), plant height (11.23%), and shoot fresh weight (20.38%) in bananas (Figure 3).

### The banana plants’ antioxidant enzymes activity responses associated with *Trichoderma koningiopsis* Tk905 inoculation

**Figure 4:**
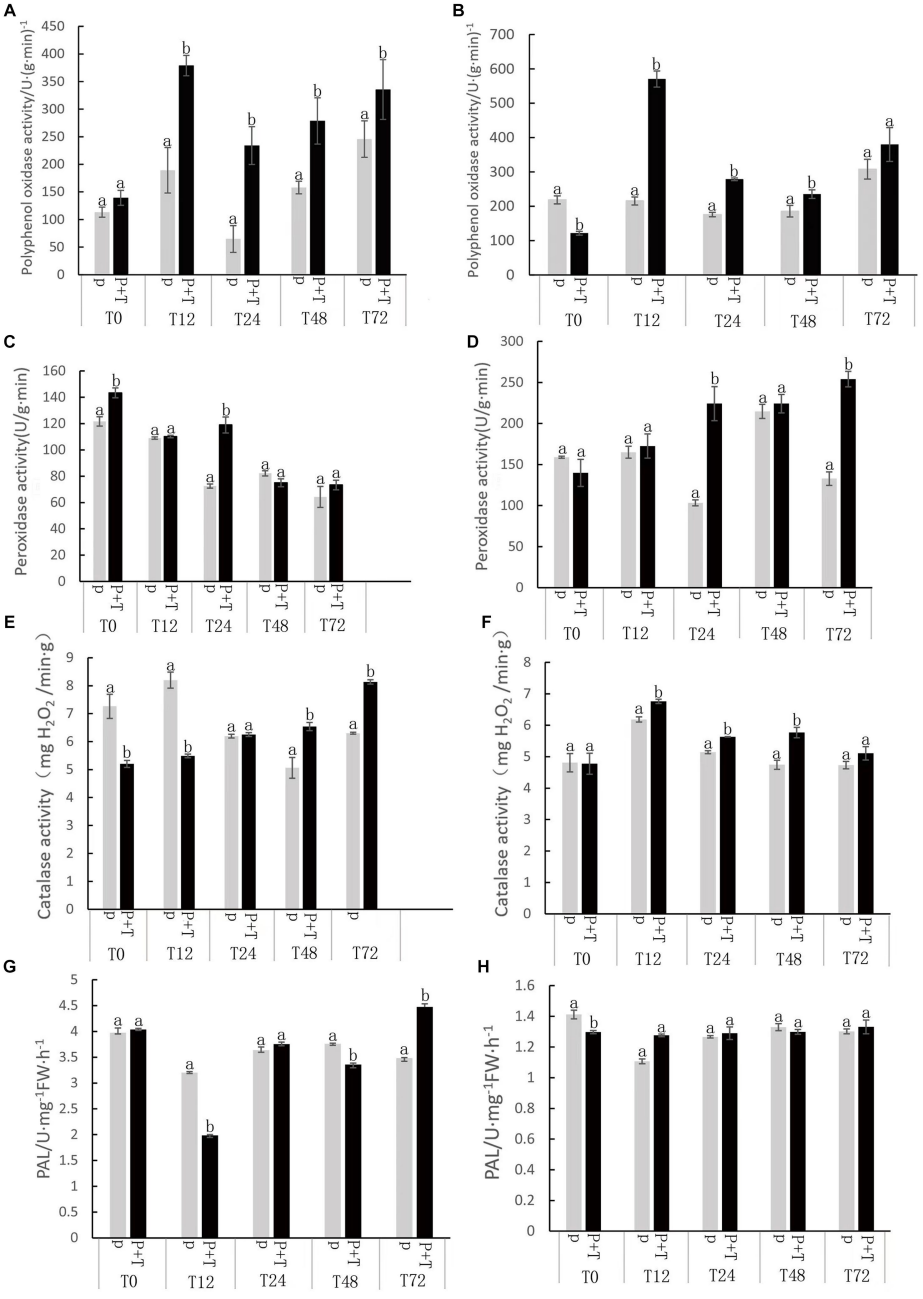
Catalase (CAT), Phenylalanine ammonia-lyase (PAL), Polyphenol oxidase (PPO), and sodium dismutase (SOD), kinetic levels as affected by various treatments including with (P) or without *Trichoderma koningiopsis* Tk905 (P + T) of 0, 12, 24, 48, and 72 h after Tk905 inoculation. The mean values in each of the columns that begin with the same letter are not statistically different (*p* ≤ 0.05). **(A,C,E,G)** from leaves, and **(B,D,F,H)** from roots.

Upon inoculation with Tk905, notable changes were observed in the activity levels of the antioxidant enzymes CAT, PAL, PPO, and SOD in both leaves and roots of the banana plants (Figure 4). Recognized for its crucial role in scavenging free radicals in plants, the activity levels of POD in the roots were consistently elevated in treated plants across all time points. Notably, a peak was observed at 72 h post-treatment. By 24 h, POD levels in treated leaves and roots were 1.63 times and 2.17 times higher than the controls, respectively. For treated plants, there was a heightened PPO activity in both leaves and roots at all monitored time points. A peak activity, 3.6 times (leaves) and 1.58 times (roots) higher than the control, was recorded 12 h post-treatment. Maximum CAT activity was reached at 72 h in leaves and 12 h in roots. The root’s CAT activity under Tk905 treatment showed a remarkable increase of 21.27% at 48 h. Playing a pivotal role in plant defense under adverse conditions, PAL activity showed an upward trend in both leaves and roots post-Tk905 treatment. The leaves registered a significant 28.7% boost in PAL activity by 72 h (Figure 4).

### Compatibility of endophytic *Trichoderma* Tk905 strain with fungicides

All the seven tested agents had inhibitory effects on FocTR4-14013, and the inhibition rate ranged between 61.61–81.04% and 57.14–79.15% 3 and 7 d after fungicide treatment, respectively. On the third day of treatment of FocTR4-14013 with seven fungicides, the highest inhibition rate was found in the water dispersible difenoconazole, which reached 81.01%, followed by the pydiflumetofen + difenoconazole suspension agent which exhibited a 79.15% inhibition rate (Table 1).

**Table 1:** Plate fungistatic tests of seven fungicides against *Fusarium oxysporum* f. sp. *cubense* tropical race 4.

Experimental treatment	3 d		7 d	
	Average colony diameter ± SD/cm	Inhibition rate/%	Average colony diameter ± SD/cm	Inhibition rate/%
CK	4.22 ± 0.01	0	7.77 ± 0.26	0
Pyraclostrobin∙Fluxapyroxad	1.61 ± 0.03	61.61^a^	3.32 ± 0.12	57.27^a^
Difenoconazole	0.8 ± 0.01	81.04^c^	1.62 ± 0.01	79.15^c^
Difenoconazole∙ Azoxystrobin	1.12 ± 0.01	73.46^b^	2.35 ± 0.02	69.76^b^
Fluopyram∙Trifloxystrobin	1.27 ± 0.19	69.90^b^	2.57 ± 0.26	66.92^b^
Mefentrifluconazole∙Pyraclostrobin	1.51 ± 0.03	64.22^a^	3.33 ± 0.02	57.14^a^
Pydiflumetofen∙Difenoconazole	0.88 ± 0.07	79.15^c^	1.72 ± 0.11	77.86^c^
Propiconazole∙Azoxystrobin	1.17 ± 0.02	72.27^b^	2.64 ± 0.02	66.02^b^

Different letters indicate differences among the same column by the Duncan’s multiple range test (*p* ≤ 0.05).

The two most effective fungicides (difenoconazole and pydiflumetofen + difenoconazole), were selected for compatibility tests with Tk905. Although the above fungicides two had some limiting effects on TK905 growth at a concentration of 50 μg/mL, it could still grow, and the inhibition rate fell to approximately 20% after 8 d. Additionally, Tk905 can grow well in concentrations of 10 μg/mL and 20 μg/mL. Overall, results suggested that *T. koningiopsis* Tk905 was compatible with difenoconazole and pydiflumetofen + difenoconazole since it could grow normally on plates containing these fungicides at the recommended doses (Table 2).

**Table 2:** Determination of compatibility between fungicide and Tk905.

Experimental treatment	5 d		8 d	
	Colony diameter/cm	Inhibition rate/%	Colony diameter/cm	Inhibition rate/%
CK	8.47 ± 0.01	–	8.4 ± 0.01	–
Difenoconazole 10 μg/mL	5.56 ± 0.06	34.36^b^	8.21 ± 0.05	2.26^a^
Difenoconazole 20 μg/mL	4.72 ± 0.09	44.27^c^	8.21 ± 0.01	2.26^a^
Difenoconazole 50 μg/mL	3.31 ± 0.04	60.92^e^	6.66 ± 0.15	20.71^b^
Pydiflumetofen∙Difenoconazole 10 μg/mL	7.97 ± 0.08	5.90^a^	8.31 ± 0	1.10^a^
Pydiflumetofen∙Difenoconazole 20 μg/mL	7.93 ± 0.07	5.90^a^	8.31 ± 0.01	1.10^a^
Pydiflumetofen∙Difenoconazole 50 μg/mL	4.08 ± 0.03	51.83^d^	6.7 ± 0.08	20.23^b^

Different letters indicate differences among the same column by the Duncan’s multiple range test (*p* ≤ 0.05).

### Impact of Tk905 on the growth of banana plants against FocTR4 under greenhouse conditions

To determine whether *T. koningiopsis* Tk905 could reduce the symptoms of *FocTR4* infection in bananas, 5-leaf stage banana plant roots were treated with Tk905 using three different treatment methods: root irrigation, incorporation in soil, and spraying leaves using sterile fermentation broth as a dispersion medium and their susceptibility to *FocTR4* (+T+F) leaf infection was subsequently assessed. MTk905-untreated plants (−T+F) and banana plants that were not treated with neither the pathogen nor the control agent (−T−F) were also included. Treatment of banana plants with Tk905 significantly reduced the disease index 45 days after inoculation (*p* < 0.05; Table 3).

**Table 3:** Effect of inoculation with *Trichoderma koningiopsis* Tk905 on the disease incidence and severity of Fusarium wilt 45 days after inoculation.

Treatments	Morbidity (%)	Disease index	Control effect (%)
−T+F	75.19 ± 5.48^a^	41.77 ± 4.88^a^	–
+T+F (Root irrigation)	46.15 ± 7.30^b^	23.59 ± 3.56^b^	43.53 ± 8.52^c^
+T+F (Mix soil)	35.87 ± 5.14^b^	19.66 ± 4.39^b^	52.92 ± 10.53^b^
+T+F (spray)	56.67 ± 14.53^ab^	25.18 ± 6.42^b^	40.61 ± 15.38^c^
+T+Pydiflumetofen∙Difenoconazole	25.06 ± 3.59^c^	17.92 ± 5.26^c^	57.10 ± 8.23^b^
+T+F(Mix soil) + Pydiflumetofen∙Difenoconazole	25.06 ± 6.56^c^	11.95 ± 5.29^d^	71.40 ± 8.75^a^
−T−F	0^c^	0^c^	–

T, Trichoderma koningiopsis Tk905; F, Fusarium oxysporum f. sp. cubense tropical race 4 strain 14,013. Different letters indicate differences among the same column by the Duncan’s multiple range test (*p* ≤ 0.05).

Among different Tk905 application methods, the banana wilt disease control effect was found to be the best in the Tk905 irrigation (8 mL) treatment group reaching 52.41%, followed by the Tk905 incorporated with soil treatment group (49.6%). Furthermore, the combination of Tk905 with the pydiflumetofen + difenoconazole fungicide resulted in a synergistic effect: 71.40% control achieved by the combination while 52.92% by Tk905 and 57.10% by pydiflumetofen + difenoconazole alone (Table 3).

## Discussion

*FocTR4* is devastating banana pathogen causing significant losses of production worldwide (Kema, 2019). Being a typical soil-borne disease, the efficient long-term control of banana Fusarium wilt remains challenging and requires the development of novel control methods. The utilization of resistant varieties, such as the Formosana banana (Hwang and Ko, 2004), is considered the most efficient, environmentally friendly, and cost-effective method for managing losses caused by Fusarium. However, no immune banana species to *FocTR4* have been identified (Hwang and Ko, 2004; Kema, 2019). Currently, this disease is controlled by the application of synthetic chemicals including fungicides and soil fumigants. However, the detrimental effects of these chemicals on the environment (Zhou et al., 2016) and the demand for green ecological agriculture have led to the evaluation of biocontrol agents (BCAs) as suitable disease control methods for banana Fusarium wilt (Fu et al., 2017; Ismaila et al., 2023).

Bacillus spp. (Sun et al., 2022), Streptomyces spp. (Li et al., 2021), and Trichoderma spp. (Vlot et al., 2021; Abdelkhalek et al., 2022) are widely used BCAs. Trichoderma spp. has been shown to activate plant systemic resistance in addition to its biocontrol attributes (Vlot et al., 2021; Abdelkhalek et al., 2022). Trichoderma was also found to act as a probiotic agent that modify the composition and function of the resident soil fungal microbiome to suppress soil-borne diseases (Tao et al., 2023).

Although *T. harzianum* is the most widely studied and used species, other species such as *T. asperellum*, *T. viride*, *T. koningii*, and *T. koningiopsis* have also been investigated for their biological control potential in different crops (Samuels et al., 2006; Diego et al., 2021; Abdelkhalek et al., 2022). *T. koningiopsis* was first described by Samuels et al. (2006). *T. koningiopsis* JA14 was isolated from cucumber planting soil in Jinagxi Province, China (Li et al., 2010). In this study, we isolated and identified the endophytic *T. koningiopsis* Tk905 strain from the roots of dendrobe plants. Morphological and molecular analyses confirmed the strain to be *T. koningiopsis*, which is widely distributed in tropical regions such as South America, East Africa, Europe, Canada, and Eastern North America (Samuels et al., 2006). *T. koningiopsis* has been reported to exhibit antagonistic activity against various plant pathogens (Li et al., 2010; Chen et al., 2015; Diego et al., 2021). In line with these findings, our results demonstrated that *T. koningiopsis* Tk905 exhibits significant antifungal activity against FocTR4 and promotes the growth of banana plants.

Niche occupation also has an important effect on microbial communities and can affect the severity of plant diseases. Pathogen resistance induced by microbial resource competition networks has recently been observed in bacterial communities (Wei et al., 2015). Furthermore, Tao et al. (2023) recently proved that the degree of niche overlap between resident fungi and pathogens plays an important role in the level of pathogen suppression. The antifungal activity of *T. koningiopsis* Tk905 was further validated by analyzing its ability to inhibit the mycelial growth of eight plant pathogens. Results revealed that Tk905 covered the pathogen mycelia, and spores were observed on the pathogen hyphae or around them. These findings suggest that *T. koningiopsis* Tk905 exerts its antifungal activity through direct antagonism and competition for nutrients and spaces, indicating that Tk905 can occupy the niche of the soil microbial community and suppress pathogens.

Recent studies have reported significant growth-promoting effects of Trichoderma application on various crops (Vlot et al., 2021; Abdelkhalek et al., 2022; Tao et al., 2023). This is in agreement with our results which demonstrated that the average root fresh weight, plant height, and shoot fresh weight of Tk905-inoculated plants were significantly higher than those of the untreated plants. Possible mechanisms underlying the observed enhanced banana plant growth by Tk905 treatment may include phytohormone production, mineral solubilization, or nutrient uptake facilitation.

The induction of plant resistance is considered to be more critical for the overall improvement of plant immunity than other biocontrol mechanisms, such as antibiosis, competition, and mycoparasitism (Cai and Druzhinina, 2021). Some *Trichoderma* spp. have been shown to activate plant immune responses by being recognized by the pattern recognition receptors (PRRs) on plant cell membranes. Our study revealed that Tk905 effectively controlled banana wilt when applied via foliar spraying and other treatments. Moreover, the application of Tk905 resulted in various changes in the activity of resistance enzymes in banana, indicating its potential to induce disease resistance in plants. Furthermore, Tk905 treatment significantly increased the activity of these antioxidant enzymes, suggesting that Tk905 may enhance plant defense systems by activating their antioxidant mechanisms.

The pronounced elevation in the activities of antioxidant enzymes in banana plants post-inoculation with *T. koningiopsis* Tk905 signifies the plant’s enhanced resistance mechanism. These enzymatic responses not only indicate a direct protective role against potential oxidative damage but also hint at the plant’s proactive measures to combat stresses. Intriguingly, the varied peak times of these enzyme activities – such as POD peaking at 72 h, PPO at 12 h, and CAT and PAL at different intervals – suggest a coordinated and synergistic action. Such a staggered yet harmonized response might be the plant’s strategic move to ensure a sustained and prolonged resistance against potential threats. This temporal synergy of enzymatic activities provides an added layer of defense, making the induced resistance likely more durable and effective. Future investigations into the molecular dialogues between banana plants and Tk905 could provide insights into the exact mechanisms driving these orchestrated enzymatic defenses.

Our results demonstrated that Tk905-treated plants exhibited significantly lower disease incidence and severity than untreated plants. This finding is consistent with previous reports of *Trichoderma* spp. suppressing Fusarium wilt in various crops (Harman et al., 2004; Sharma et al., 2013; Tao et al., 2023). Notably, the protective effects of Tk905 against FocTR4 infection were not only observed in the early stages of infection but also persisted throughout the experiment, suggesting that Tk905 provides a long-lasting protection against Fusarium wilt.

## Conclusion

In summary, our findings demonstrated that *T. koningiopsis* Tk905 possesses significant anti-FocTR4 activity, induced resistance to FocTR4, and promotes growth in banana plants. This study provides valuable insights on improving biological control strategies against FocTR4 infection in bananas. However, further research is needed to explore the biological control mechanisms of Tk905 and its effectiveness under different environmental conditions. Moreover, considering the various challenges that biological control agents may face in large-scale applications (such as environmental adaptability, persistence, and production costs), combining Tk905 with other biological control agents or integrated management strategies may further improve its effectiveness in practical applications.

## Data availability statement

The datasets presented in this study can be found in online repositories. The names of the repository/repositories and accession number(s) can be found at: https://www.ncbi.nlm.nih.gov/genbank/, OR083438.

## Author contributions

ML: Conceptualization, Formal analysis, Funding acquisition, Methodology, Project administration, Resources, Validation, Writing – original draft, Writing – review & editing. YC: Data curation, Investigation, Methodology, Resources, Writing – review & editing. QH: Data curation, Software, Writing – review & editing. ZH: Investigation, Methodology, Writing – review & editing. HS: Formal analysis, Writing – review & editing. ZD: Conceptualization, Funding acquisition, Project administration, Validation, Writing – review & editing, Writing – original draft.

## Footnotes

^1^ https://www.ncbi.nlm.nih.gov/

^2^ https://www.ebi.ac.uk/Tools/msa/mafft/
